# Diagnostic Molecular Markers for Phosphine Resistance in U.S. Populations of *Tribolium castaneum* and *Rhyzopertha dominica*

**DOI:** 10.1371/journal.pone.0121343

**Published:** 2015-03-31

**Authors:** Zhaorigetu Chen, David Schlipalius, George Opit, Bhadriraju Subramanyam, Thomas W. Phillips

**Affiliations:** 1 Department of Entomology, Kansas State University, Manhattan, Kansas, United States of America; 2 Department of Agriculture, Fisheries and Forestry, Agri-Science Queensland, Brisbane, Queensland, Australia; 3 Department of Entomology and Plant Pathology, Oklahoma State University, Stillwater, Oklahoma, United States of America; 4 Dept. of Grain Science and Industry, Kansas State University, Manhattan, Kansas, United States of America; 5 Plant Biosecurity Cooperative Research Center, Bruce, Australian Capital Territory, Australia; Federal University of Viçosa, BRAZIL

## Abstract

Stored product beetles that are resistant to the fumigant pesticide phosphine (hydrogen phosphide) gas have been reported for more than 40 years in many places worldwide. Traditionally, determination of phosphine resistance in stored product beetles is based on a discriminating dose bioassay that can take up to two weeks to evaluate. We developed a diagnostic cleaved amplified polymorphic sequence method, CAPS, to detect individuals with alleles for strong resistance to phosphine in populations of the red flour beetle, *Tribolium castaneum*, and the lesser grain borer, *Rhyzopertha dominica*, according to a single nucleotide mutation in the dihydrolipoamide dehydrogenase (DLD) gene. We initially isolated and sequenced the DLD genes from susceptible and strongly resistant populations of both species. The corresponding amino acid sequences were then deduced. A single amino acid mutation in DLD in populations of *T*. *castaneum* and *R*. *dominica* with strong resistance was identified as P45S in *T*. *castaneum* and P49S in *R*. *dominica*, both collected from northern Oklahoma, USA. PCR products containing these mutations were digested by the restriction enzymes MboI and BstNI, which revealed presence or absence, respectively of the resistant (R) allele and allowed inference of genotypes with that allele. Seven populations of *T*. *castaneum* from Kansas were subjected to discriminating dose bioassays for the weak and strong resistance phenotypes. Application of CAPS to these seven populations confirmed the R allele was in high frequency in the strongly resistant populations, and was absent or at a lower frequency in populations with weak resistance, which suggests that these populations with a low frequency of the R allele have the potential for selection of the strong resistance phenotype. CAPS markers for strong phosphine resistance will help to detect and confirm resistant beetles and can facilitate resistance management actions against a given pest population.

## Introduction

Cereal grains can be stored for a year or more following harvest before they are milled for food and feed, during which time they are subject to infestation by various species of stored-product pests [1]. Hydrogen phosphide gas (PH_3_), known commonly as phosphine, is an effective and widely used pesticide for disinfestation of stored grains, but insects in many countries have developed resistance to this toxin. The red flour beetle, *Tribolium castaneum* (Herbst) (Coleoptera: Tenebrionidae) and the lesser grain borer, *Rhyzopertha dominica* (F) (Coleotpera: Bostrichidae) are two of the most destructive stored-product insect pests worldwide as they cause substantial economic loss to stored cereal grains and grain products each year. Phosphine resistant populations of *T*. *castaneum* and *R*. *dominica* have been reported in many places worldwide, presumably due to selection for resistance by long-term and sub-optimal fumigation with phosphine for management of stored product pests [2–8]. For example, in 1990 Zettler and Cuperus [3] reported using discriminating dose bioassays and showed that 13% (1 out of 8) of *T*. *castaneum* populations and 67% (8 out of 12) of *R*. *dominica* populations in grain storage areas of Oklahoma in the USA were resistant to phosphine. Twenty-five years later Opit et al. [8] sampled insects from these same areas and found that phosphine resistance frequencies had increased to 89% for populations of *T*. *castaneum* and 100% for *R*. *dominica*. Opit et al. [8] also used laboratory susceptible populations of these pest species to compute resistance ratios for the toxicity of phosphine for resistant populations compared to susceptible populations, based on the estimated lethal concentration to kill 99% of the sampled population (the LC_99_) following dose-mortality experiments. They found that the most resistant *T*. *castaneum* population required a phosphine dose for control that was 119-fold greater than that needed for susceptible beetles, and that the most resistant *R*. *dominica* population required a phosphine dose that was 1520-fold higher than that for susceptible insects.

Detection of resistant individuals in populations of insects presumed to harbor phosphine resistance is conducted with a discriminating dose bioassay described nearly 40 years ago [9] for several species of grain pests. These so-called FAO bioassays use a discriminating dose of phosphine against adult beetles of a given species that is slightly above the LC_99.9_ concentration determined for susceptible insects when exposed for 20 hrs at 25^o^ C; any test insect that survives the discriminating dose after a 14-day recovery period is deemed resistant. Research in Australia during the past decade has reported two resistant phenotypes for *T*. *castaneum* and *R*. *dominica*: beetles with “weak” resistance that may require phosphine concentrations of 10-fold to 50-fold greater than those needed to kill susceptible beetles, and beetles with “strong” resistance that may require 100-fold or greater concentrations relative to susceptible conspecifics to achieve LC_99.9_ levels of mortality. These two phosphine-resistance phenotypes were reported to have high levels of heritability involving two gene loci that mediate expression of the resistance phenotypes in both *R*. *dominica* and *T*. *castaneum* [10–13].

Identity of the gene responsible for the strong resistance phenotype in both *T*. *castaneum* and *R*. *dominica* from Australia was reported recently by Schlipalius et al. [14] as the metabolic enzyme dihydrolipoamide dehydrogenase, or DLD. This group reported several independently arising point mutations in the DLD gene, each resulting in a single amino acid change contributing to strong phosphine resistance in *T*. *castaneum* and *R*. *dominica*, as long as these insects were also homozygous for phosphine resistance alleles at a separate locus found to be responsible for weak resistance. This discovery, that only one of a small number of point mutations conferred strong phosphine resistance, allowed development of simple molecular methods to detect individuals of these species carrying strong-resistance genes for phosphine. A cleaved amplified polymorphic sequence (CAPS) [15] marker assay was developed to detect strong phosphine resistance in populations of *R*. *dominica* from Queensland in Australia [16]. Although Opit et al. [8] found resistance levels in USA populations of *T*. *castaneum* and *R*. *dominica* that could be assigned to the strong resistance phenotype, there has been no confirmation that strong resistance to phosphine in the USA is related to the same point mutation(s) in the DLD gene reported from Australia. The objectives of research reported here were to: 1) determine if point mutations exist in the coding sequence of DLD gene from susceptible and strong resistance phenotypes in populations of *T*. *castaneum* and *R*. *dominica* in the USA by sequencing the DLD gene and aligning the sequences with those of previously published susceptible and resistant strains of both species; 2) develop a CAPS molecular marker for strong phosphine resistance based on fixed resistance mutations in populations of *T*. *castaneum* and *R*. *dominica*, and 3) compare resistance levels among field populations of *T*. *castaneum* using discriminating dose bioassays for weak and strong resistance and compare these results with the frequency of a CAPS diagnostic for strong resistance in these same pest populations.

## Materials and Methods

### Insects

Beetles used in sequencing and CAPS marker development, except where noted, were laboratory reared for up to 10 generations and derived from the same populations collected and reported by Opit et al. [8], who also elaborated collection details. No specific permission from any government agency or relevant regulatory body was required for the collection of these insects at all of the sites listed below. Most collection sites were private farms that had insect-infested stored grain on site and for which the owners gave us full permission to collect insects. Other collection sites were university research farms for which we had full permission to collect insects. None of the field sites or the collections of insects from them involved endangered or protected species. *R*. *dominica* were reared on a mixture of 95% whole-wheat kernels and 5% cracked kernels admixed (wt:wt), and *T*. *castaneum* were reared on a mixture of 95% all-purpose wheat flour and 5% Brewer’s yeast (wt:wt). Both species were reared in an incubator at 28°C and 65% relative humidity with a photoperiod of 16 hrs light and 8 hrs dark. *R*. *dominica* were from the following locations with given population names. We used three Oklahoma populations from the counties of Garfield (RdOK-G), Logan (RdOK-L) and Payne (RdOK-P) with a fourth population collected by us during 2012 in stored wheat from the state of Georgia near the town of Tifton (RdGA-T). *T*. *castaneum* were also from the same three Oklahoma counties of Garfield (TcOK-G), Logan (TcOK-L) and Payne (TcOK-P). Oklahoma samples for each species were originally collected from single steel bins of wheat (Payne County) or single concrete silos of wheat (Garfield and Logan counties) using WB-II pitfall probe traps deployed for at least 7 days that capture adult beetles moving in the top 30 cm of grain [8]. The Georgia sample of *R*. *dominica* came from adults emerging from open 400 ml cups of grain deployed at one location to sample flying insects over several days. Field collected insects were from bins and silos or grain storage areas with grain bins (i.e., the Georgia beetles) that were treated yearly with phosphine fumigation. For each species we also studied a phosphine-susceptible laboratory population (RdLab-S and TcLab-S) that were from laboratory colonies maintained in isolation by the USDA ARS in Manhattan, KS for more than 40 years, the ancestors of which were very likely to have never experienced phosphine fumigation.

### Amplification of DLD gene from *T*. *castaneum* and *R*. *dominica*

#### Total RNA and poly(A) RNA isolations

Total RNA was isolated from 25 individuals from each strain of *T*. *castaneum* and *R*. *dominica*, respectively using Trizol reagent (Life Technologies, Gaithersburg, MD, USA). Poly(A) RNA was purified from total RNA using RevertAid First Strand cDNA Synthesis Kit according to the manufacturer’s instruction (Thermo Scientific, Waltham, MA, USA).

#### DLD gene from *T*. *castaneum* and *R*. *dominica*

The DLD gene from both susceptible and resistant populations of *T*. *castaneum* and *R*. *dominica* was amplified using cDNA as a template in 25μl reaction volume. The mixture included 12.5μl Master Mix, 1μl each corresponding forward and reverse primers at concentrations of 10.0 μmole (S1 Table), 2μl cDNA template (at approximately 50 ng/μl) and 8.5μl ddH_2_O using Thermo Scientific PCR MasterMix polymerase kit. The PCR temperature program was the following: denaturation at 95°C for 5min; 36 cycles at 95°C for 15s, 58°C (55°C for *R*. *dominica*) for 30s and 72°C for 2min for denaturation, annealing and extension, respectively; and a final extension at 72°C for 10min. In order to obtain the whole coding sequence of the DLD gene from *R*. *dominica*, additional PCRs were set up with the following primer combinations, described in detail in S1 Table: Rd-F with Rd-In-R and Rd-In-F with Rd-R. To sequence the DLD coding sequence of *T*. *castaneum*, only the Tc-In-F and Tc-In-R (S1 Table) primers were required. PCR products were purified from the gels using QIAEX II Gel Extraction Kit according to manufacturer’s instruction (QIAGEN Science, Maryland, USA). The purified PCR products were sequenced directly using ABI 3700 DNA Sequencer at the Kansas State University DNA Sequencing Facility (Manhattan, KS USA). The whole lengths of cDNA of DLD from *T*. *castaneum* and *R*. *dominica* were assembled from the sequence fragments using DNAStar software and the deduced amino acid sequences were obtained using the www.expasy.com website. Nucleotide sequences and deduced amino acid sequences from susceptible and resistant populations of *T*. *castaneum* and *R*. *dominica* were aligned against Australian populations using Clustal W2 [17].

### Detection of phosphine resistance alleles in *T*. *castaneum* and *R*. *dominica*

#### Extraction of Genomic DNA

Genomic DNA was extracted from 16 individuals from the resistant populations of each species, TcOK-G and RdOK-G, using a Chelex-100 DNA extraction method as described by Schlipalius et al. (2012) with slight modification for improved product as follows. Briefly, an individual beetle was homogenized in 200μl 10% Chelex-100 and boiled for 20min. Then samples were placed onto ice for 2min. Samples were centrifuged at 5000 rpm at room temperature for 10min. Aliquots of each sample were diluted 20X for the subsequent PCR.

### Development of a resistance marker

A cleaved amplified polymorphic sequence (CAPS) marker assay was designed to target a SNP (single nucleotide polymorphism) found in the DLD gene sequences of TcOK-G and RdOK-G. A fragment from each of the relevant gene sequences was amplified in 25μl reaction volume (12.5μl Master Mix, 1μl each forward and reverse primer, 2μl gDNA template and 8.5μl ddH_2_O) using Thermo Scientific PCR MasterMix polymerase kit as described earlier. Primers Tc-MM-F paired with Tc-MM-R and Rd-MM-F paired with Rd-MM-R (see S1 Table) were used in the PCRs for *T*. *castaneum* and *R*. *dominica*, respectively (S1 Table)**.** The PCR program was set as the following: denaturation at 95°C for 5min; 30 cycles at 95°C for 15s, 58°C (55°C for *R*. *dominica*) for 30s and 72°C for 2min for denaturation, annealing and extension, respectively; and a final extension at 72°C for 10min. The amplified 368bp PCR product from *T*. *castaneum* and the 375bp PCR product from *R*. *dominica* were subjected to separate restriction enzyme digestion with either MboI or BstNI (New England Biolabs, MA USA) in a 10μl reaction containing 8μl of PCR product, 1μl reaction buffer and 1U restriction enzyme. The reaction was then incubated at 37°C for MboI or 60°C for BstNI, respectively for 15min according to manufacturer’s instruction.

### Association of CAPS markers for phosphine resistance genotypes in *T*. *castaneum* and *R*. *dominica* with resistance phenotypes determined from bioassay

In order to confirm that the CAPS markers we developed could be used to score genotypes and gene frequencies for a strong resistance allele in populations that had known frequencies of phosphine resistance phenotypes, we first conducted discriminating dose bioassays for four *T*. *castaneum* populations and five *R*. *dominica* populations, with 20 adult beetles from each population. We used FAO method No.16 [9] with phosphine concentrations of 30 ppm for *T*. *castaneum* and 20 ppm phosphine for *R*. *dominica*, each exposed for 20 hrs at 25°C and followed by a 14-day period for either recovery or delayed mortality before assigning an individual as either resistant (alive) or susceptible (dead). Quantification and application of phosphine concentrations for bioassays were conducted as in Opit et al. [8].

### Bioassay determination of resistance in field populations of *T*. *castaneum* followed by CAPS marker analysis

**Fig 1:**
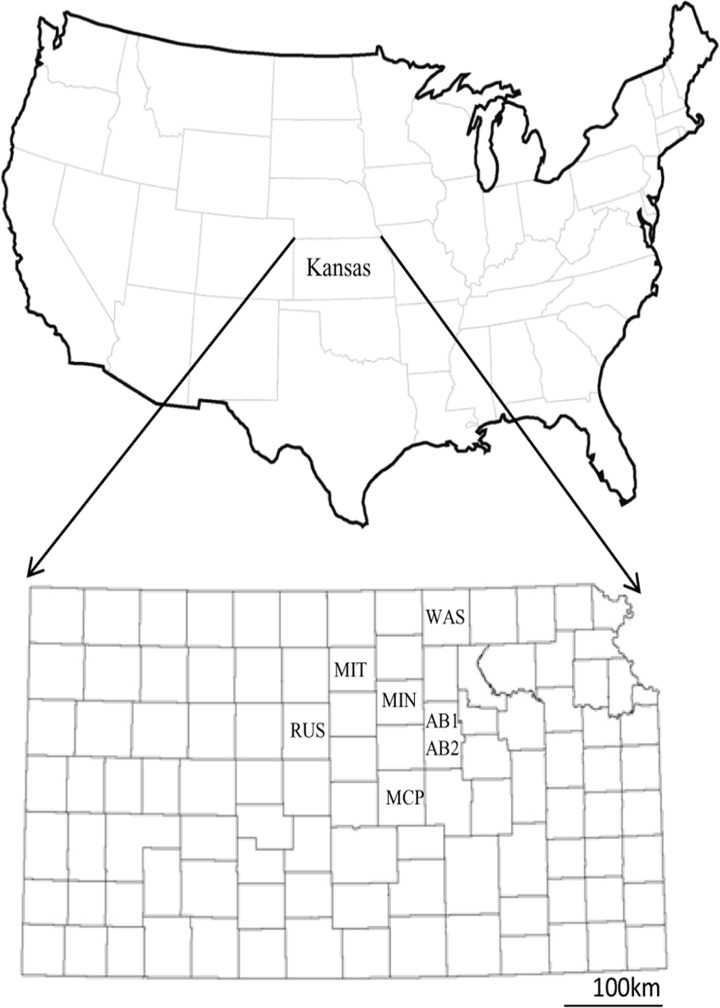
Locations for field-collected populations of *T*. *castaneum* in Kansas, USA.

An opportunity presented itself for us to compare the frequency of the resistance gene in *T*. *castaneum* with the percentage of resistant insects in seven locally collected populations. *T*. *castaneum* were collected in 2011 from seven farms that had stored wheat on site, all within a 100 km radius of each other, in north-central Kansas, USA (Fig 1) and maintained as large laboratory-reared colonies until used [18]. The approximate geographic locations of the collection sites, by population codes for latitude and longitude, respectively, are: AB1, 39°01′ N and 97°12 W; AB2, 38°59′N and 97°19′W; MCP, 38°22′N and 97°39′W; MIT, 39°20′N and 98°28′W; MIN, 39°07′N and 97°42′W; RUS, 38°53′N and 98°51′W; and WAS, 39°34′N and 97°16′W. In order to score both weak and strong phosphine resistance in these populations we carried out a modified FAO (1975) bioassay with 50 individuals/vial and 3 replicates per population. The discriminating dose for weak resistance was 30ppm for 20 hrs, as described above, and the discriminating dose for the strong resistance phenotype was set at 180ppm for 20 hrs [19] in separate experiments. Molecular marker analysis for the strong resistance SNP marker was performed on all seven populations as described above and its frequency was calculated for each population from the individual genotypes inferred by the CAPS markers.

## Results

### cDNA sequences from susceptible and resistant U.S. populations of *T*. *castaneum* and *R*. *dominica*

**Fig 2:**
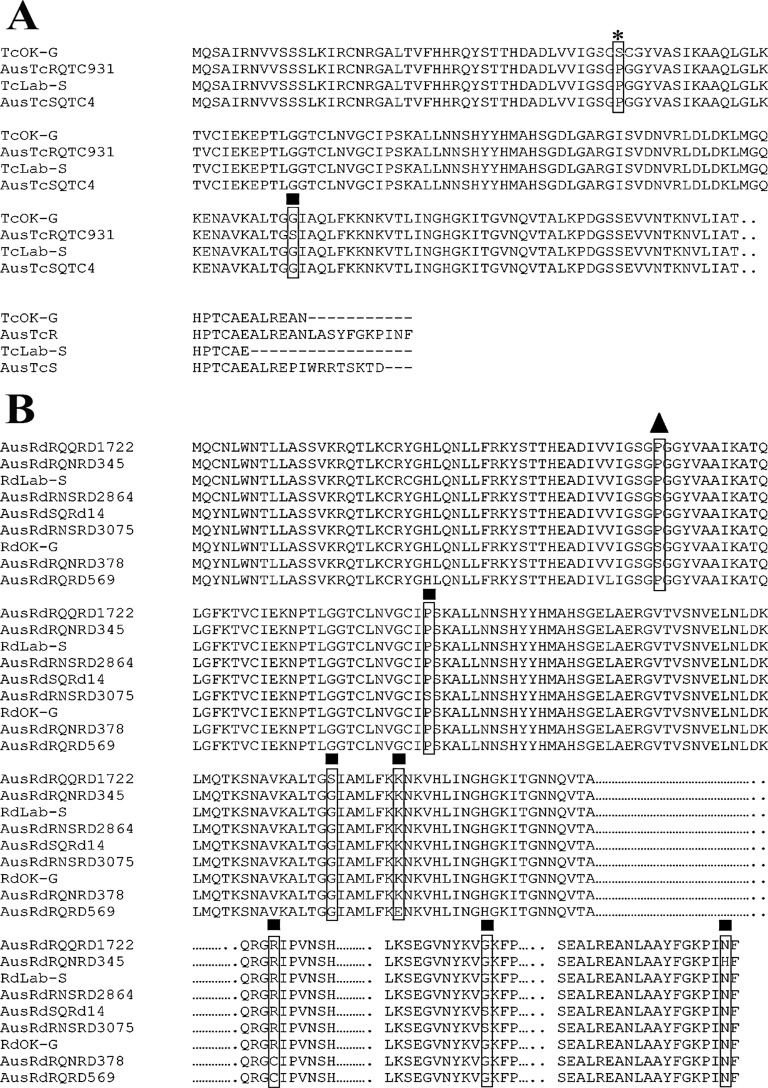
Deduced amino acid sequences of DLD from *T*. *castaneum* and *R*. *dominica* by ClustalW2. Only the portions of the amino acid sequences with relevant mutations in both species are shown. **Fig 2A:** Sequences from susceptible and resistant strains of *T*. *castaneum* from the USA aligned against Australian strains. **Fig 2B:** Sequences from susceptible and resistant strains of *R*. *dominica* from the USA aligned against Australian strains. Differences between the phosphine-susceptible and phosphine-resistant sequences are boxed. The asterisk denotes the P45S phosphine-resistance mutations described in the *T*. *castaneum* population TcOK-G. The triangle denotes the P49S phosphine-resistance mutations described in the text. The squares denote all the other phosphine-resistance mutations described only in Australian strains.

DLD gene sequences of 1600 bp and 1800bp were amplified using the first strand cDNA as templates from *T*. *castaneum* and *R*. *dominica*, respectively. In order to obtain the whole sequence of the DLD gene from *R*. *dominica*, additional PCRs were set up as described in the Materials and Methods section. DLD gene fragments of 1,100 bp and 900bp were amplified from both susceptible (RdLab-S) and resistant (RdOK-G) populations. The deduced amino acid sequences of susceptible and resistant populations of both *T*. *castaneum* and *R*. *dominica* were obtained using the www.expasy.com website after the nucleotide sequences were assembled under DNAStar software. Furthermore, amino acid sequences of susceptible and resistant *T*. *castaneum* (TcLAB-S and TcOK-G) and susceptible and resistant *R*. *dominica* (RdLab-S and RdOK-G) were aligned against those of Australian populations of susceptible and resistant *T*. *castaneum* (AusTcSQTC4 and AusTcRQTC931) and one susceptible and seven resistant *R*. *dominica* populations (AusRdSQRd14, AusRdRQQRD1722, AusRdRQNRD345, AusRdRNSRD2864, AusRdRNSRD3075, AusRdRQNRD378 and AusRdRQRD569; accession number GF111942 of ENA) studied by Schlipalius et al. [14]. A single amino acid mutation in DLD, reported by Schlipalius et al. [14] as contributing to strong phosphine resistance, was identified as P45S in *T*. *castaneum* from the TcOK-G population (labeled USTcROKG in Fig 2A) and P49S in *R*. *dominica* from the RdOK-G population (labeled USRdROKG in Fig 2B).

### Scoring phosphine resistance genotypes in *T*. *castaneum* and *R*. *dominica* with CAPS markers

**Fig 3:**
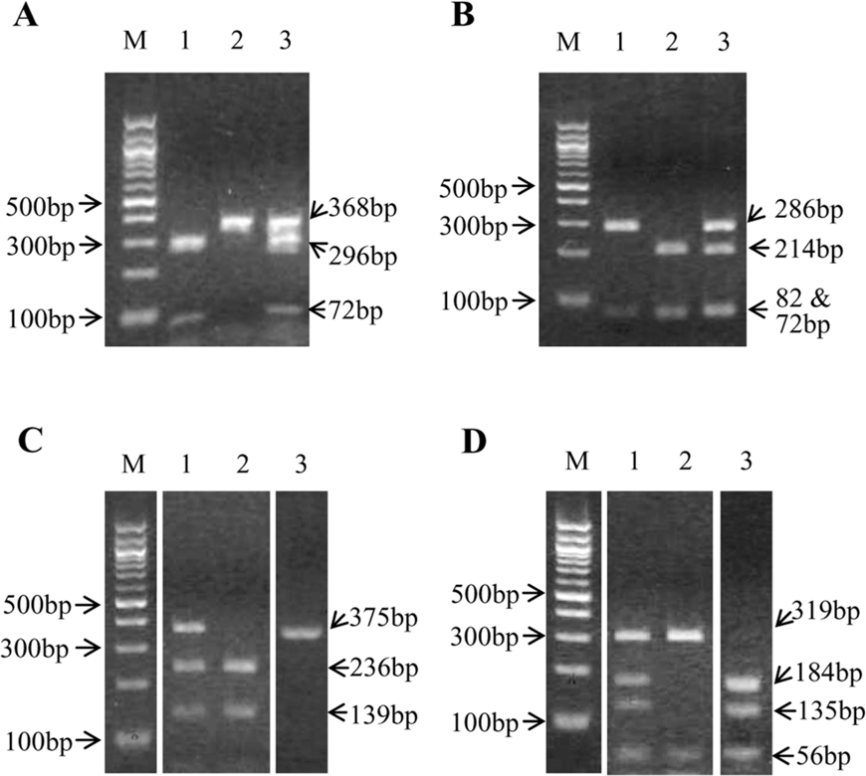
Restriction enzyme digests of PCR products amplified from genomic DNA coding for the DLD gene. M = size marker (100bp DNA Ladder, New England BioLabs Inc., MA USA); lanes 1, 2 and 3 on each gel are digests of individual beetles as noted. Gels A and B are digested products from homozygous resistant (RR), susceptible (SS) or heterozygous (RS) *T*. *castaneum* from the TcOK-G population digested with either MboI or BstNI, respectively. Gels C and D are similarly digested products from *R*. *dominica*. See text for details.

Further analysis of the DNA sequences revealed that the single P to S amino acid mutation in the DLD enzyme responsible for strong resistance in *T*. *castaneum* and *R*. *dominica* was due to a single common nucleotide change of C133T in the coding sequence of *T*. *castaneum* and a C145T change in *R*. *dominica*. CAPS markers to detect these SNPs in the two beetle species were then developed based upon the nucleotide variations observed. DNA extracted from one individual each of resistant *T*. *castaneum* (TcOK-G) and resistant *R*. *dominica* (RdOK-G) using 10% Chelex were used as templates for PCR to generate products (368bp and 375bp, respectively) that contained the SNP of interest for the CAPS analysis. These PCR products were then each digested by the MboI restriction enzyme, which recognized the target mutation site conferring the C to T for the resistance allele and cleaved the amplified sequence. In order to validate the MboI digestions, the same PCR products were also digested by BstNI restriction enzyme, which cleaved the amplified sequence at the non-mutated site 133 in phosphine susceptible (or weakly resistant) individuals (Fig 3).

Examples of restriction enzyme digests used to derive individual genotypes are shown in the gels depicted in Fig 3, in which gels A and B show *T*. *castaneum*, gels C and D show *R*. *dominica* and digests with MboI are in gels A and C while digests with BstNI are gels B and D. In the TcOK-G population, digestion with MBoI revealed 2 fragments (72 and 296bp), the original non-digested PCR product of 368bp, or 3 fragments (72, 296 and 368bp), as in Fig 3A, when the individual beetle was either homozygous resistant (RR; beetle no. 1), homozygous susceptible (SS; beetle no. 2), or heterozygous for the strong resistance allele (RS; beetle no. 3). Digestion of the same PCR products from these three *T*. *castaneum* beetles with BstNI (Fig 3B) to detect the non-mutated C at nucleotide location 133 revealed either two fragments (82 and 286bp), 3 fragments (72 unresolved from 82 and 214bp) or 4 fragments (72 unresolved from 82, 214 and 286bp) confirming the individual beetles were homozygous resistant (RR), homozygous susceptible (SS) and heterozygous resistant (RS) for beetle nos. 1, 2 and 3, respectively. In the RdOK-G population, digestion with MboI revealed 2 fragments (139 and 236bp) or 3 fragments (139, 236 and 375bp) if the individual beetle was either homozygous resistant (RR; beetle no. 2) or heterozygous resistance (RS; beetle no. 1), respectively, and a fully susceptible beetle from our RdLab-S population (beetle no. 3) had undigested 375 bp product denoting an SS genotype (Fig 3C). After digestion of product from the same RdOK-G beetles with BstNI (Fig 3D) we found 2 fragments (56 and 319bp) for the RR beetle (no. 2), 4 fragments (56, 319, 135 and 184 bp) for the RS beetle (no. 1) and the SS beetle from RdLab-S had three fragments (184, 135 and 56). BstNI recognized at least two non-target restriction sites in both species, which resulted in multiple bands beyond those needed for the intended diagnosis of their resistance genotype. These studies clearly show that MboI digests for the SNP in the DLD sequence that represents a strong phosphine resistance allele in both species can be used to assign resistance genotypes, and that the absence of the resistance allele was validated with the BstNI digests for the non-mutated target sequence.

### Strong resistance allele frequencies in phosphine-resistant strains of *T*. *castaneum* and *R*. *dominica*

Individual genotypes, and population allele frequencies, derived from the MboI CAPS markers for four populations of *T*. *castaneum* and five populations of *R*. *dominica* with bioassay-characterized resistance frequencies are summarized in Table 1. The RdOK-P population was found to be 100% resistant using the FAO assay, and all 16 beetles subjected to CAPS were homozygous resistant, whereas all RdGA-T beetles died after treatment with the FAO discriminating dose and none of the 16 beetles subjected to CAPS possessed a strong resistance allele. This RdGA-T population was identical to our susceptible RdLab-S population with regard to both phenotype and genotype. TcOK-P population had only 18% resistant individuals and none of the 16 beetles subjected to CAPS possessed the resistance allele. Results show that for this sub-set of *T*. *castaneum* and *R*. *dominica* populations studied by Opit et al. (2012), those with high resistance frequencies (above 90%) from the FAO assay also had individuals carrying the allele for strong phosphine resistance, while those with very low or zero resistance frequency (the two lab-susceptible strains and the one TcOK-P field population) probably lack the strong resistance allele in their gene pools.

**Table 1:** Genotype frequencies derived from CAPS marker analysis in *R*. *dominica* (Rd) and *T*. *castaneum* (Tc) populations.

Population*R*. *dominica*	% Resistant in FAO assay*	Strong R Genotype of DLD RR RS SS			R frequency (%)	S frequency (%)
RdOK-G	97	6	10	0	69	31
RdOK-L	90	9	7	0	78	22
RdR-OK-P	100	16	0	0	100	0
RdGA-T	0	0	0	16	0	100
RdS-Lab	0	0	0	16	0	100
*T*. *castaneum*						
TcOK-G	90	3	10	3	50	50
TcOK-L	93	0	10	6	31	69
TcOK-P	18	0	0	16	0	100
TcS-Lab	0	0	0	16	0	100

*Resistance frequencies determined from two or more samples of 20 adult beetles from each population subjected to FAO discriminating dose bioassays (*R*. *dominica* at 20 ppm PH_3_ for 20 hrs; *T*. *castaneum* at 30 ppm PH_3_ for 20 hrs).

### CAPS analysis for resistance alleles in field populations of *T*. *castaneum* from Kansas

**Fig 4:**
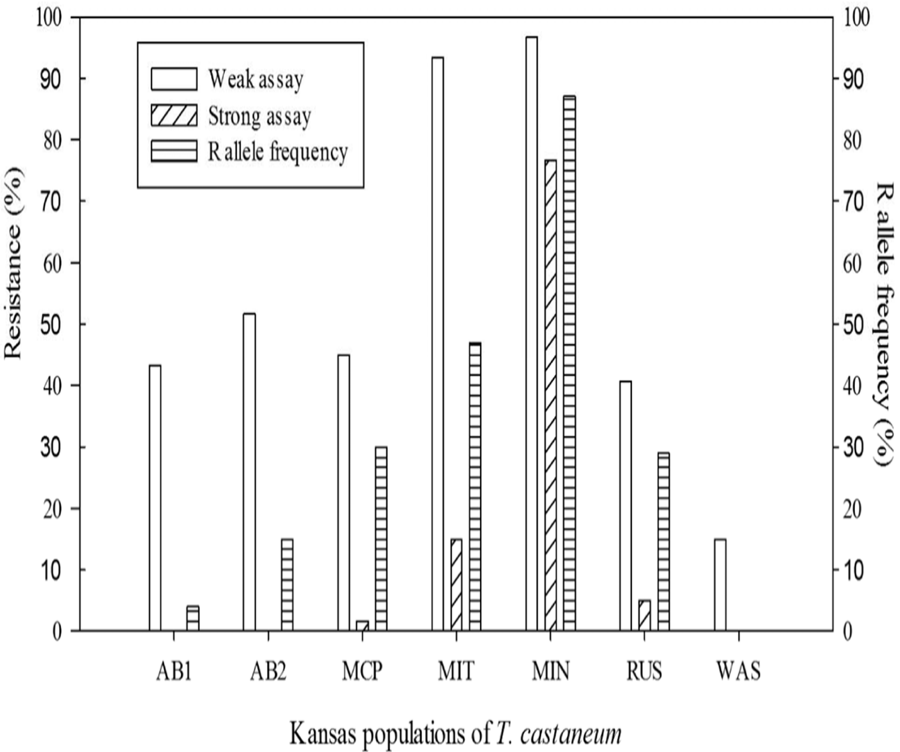
Frequency of phosphine resistance phenotypes in seven Kansas population of *T*. *castaneum*. Phenotypes were evaluated using discriminating dose bioassays for weak resistance (30 ppm phosphine for 20 hrs) and strong resistance (180 ppm for 20 hrs). The percent surviving the bioassay for a given population is equivalent to the percent resistant reported here. Also shown are the CAPS results for the frequency of the strong resistance allele in each population.

All seven populations of *T*. *castaneum* from a relatively localized geographic area of Kansas were resistant at some level to phosphine in the FAO (= weak resistance) bioassay using a 30 ppm discriminating dose, whereas four out of the seven populations were found to have beetles strongly resistant to phosphine in the bioassay using the 180 ppm discriminating dose (Fig 4). The MIN population had the highest resistance frequency (>80%) in the strong bioassay and it also had the highest R allele frequency (~87%). Alternatively, the WAS population had the lowest resistance frequency using the weak bioassay, no strong resistance was detected using the strong bioassay, and there were no R alleles detected with the CAPS procedure.

## Discussion

This work confirms that point mutations in the DLD gene are associated with strong resistance to phosphine in the beetle species *T*. *castaneum* and *R*. *dominica*, that these resistance genes occur in U.S. populations of these two species, thus increasing the geographic extent of this resistance from Australia [14] and India [20]. Our findings also point to the highly conserved nature of DLD-based phosphine resistance in grain insects, such that these same resistance alleles have independently co-evolved in totally unrelated species under the selection of phosphine use in stored grain. For *T*. *castaneum* we found only a single resistance SNP for an amino acid change of P to S (P45S) that was unknown in Australia though common in India, while the only isolated Australian resistance allele for *T*. *castaneum* [14] was for a G to S (G131S) SNP at a different position in the DLD gene. For *R*. *dominica* we found only the P49S variant of DLD, homologous to the P45S variant in *T*. *castaneum*, but none of the other four variants known for *R*. *dominica* from Australia.

The CAPS markers developed here provide straightforward information for determining the genotype of an individual at the SNP that confers strong resistance and can confirm or help predict the evolution of strong resistance in a pest population. If an appropriate number of beetles from a given population are analyzed for genotypes, then one can easily derive allele frequencies as we have done. However, our CAPS assay is specific to just one resistance allele and occurrence of additional resistance alleles unbeknownst to us at the same locus, for which there are presently no markers for detection, could cause poor inferences about observed or expected strong resistance phenotypes in a given population. Nevertheless, within the context of the populations studied here we scored genotypes from CAPS with the proposed two-allele locus in each species. Although the derived allele frequencies were not always as expected under Hardy Weinberg equilibrium, such frequencies are not unreasonable given the amount of inbreeding, drift and selection that can occur in laboratory populations. The frequency of strongly resistant individuals in a given population could be predicted from the frequency of the strong resistance allele. A low frequency or total lack of the strong resistance allele was associated with populations being scored as totally or majority susceptible to phosphine in the low dose FAO assay, whereas populations having a majority of the beetles being homozygous or heterozygous for the strong resistance allele also had a very high resistance frequency in the bioassay. Further, when we studied field populations of *T*. *castaneum* that had been in laboratory culture for less than two years, there was geographic variation in frequencies of weak and strong resistance phenotypes, and the frequency of the R allele derived from CAPS applied to these populations closely followed the frequency of strong resistance levels determined from bioassay. Interestingly, CAPS revealed that strong resistance alleles occurred at low frequency in some of these local populations of *T*. *castaneum* for which our bioassay detected zero to very few strongly resistant phenotypes, which suggests that CAPS detection of a resistance allele at the strong resistance locus might foretell the potential for a given population to evolve strong resistance phenotypes given high selection pressures under phosphine fumigation and the presence of increased resistance gene frequencies at the locus for weak resistance.

The ability to detect strong resistance genotypes in both *R*. *dominica* and *T*. *castaneum* with CAPS markers may have significance for phosphine resistance management of pest populations. The technique is relatively easy to perform, inexpensive and can be readily applied by research laboratories with a basic infrastructure for molecular techniques. The ability to use a PCR marker in the study of phosphine resistance dynamics opens many opportunities for future work not afforded by the standard FAO-type bioassays or the so-called “quick tests” for weak and strong resistance [19, 21]. Phosphine bioassays require living adults recently collected from the population of interest, access to quantitative analytical chemistry and handling of dangerous phosphine gas by researchers, which are severe limitations. Additionally, bioassays give information about the presence or absence of phosphine resistant phenotypes in a population, without any specific information on the genetic basis of the resistance. A PCR marker for DLD resistance alleles gives information on the genotypes of individual beetles and thus accurate allele frequencies in a sampled population, especially in populations where the alleles are at low frequency and so the strong resistance phenotype is undetectable. Allele frequency information allows one to assess the potential for evolution of the strong resistance phenotype in a pest population and is an early warning for the need to take a particular pest management action. CAPS can be applied to living, dead, preserved or dried specimens of beetles so that resistance gene frequency data can be obtained for collections of *R*. *dominica* and *T*. *castaneum* made over large geographic areas and at many sampling times within and across years.

An increasing number of phosphine resistant grain insect pest populations are being reported worldwide, such as in the United States, Australia, Brazil and India [7 and 8, 16, 20]. For each of these major continental areas it will be very useful to know the geographic extent of resistance and the relative strength of resistance, weak vs strong, at different locations. Rapid detection of phosphine resistance genes to help manage resistance should be aimed at identifying weak vs strong resistant populations, whereby each type could be managed differently to properly conserve the use of phosphine while also adequately controlling pest populations. Populations with no strong resistance alleles, but with evidence for weak resistance, can be controlled with future applications of phosphine that eliminate resistant insects by increasing gas concentrations with longer exposure times, warmer temperatures and in well-sealed structures, as has been the case for many Western Australian populations of *R*. *dominica* [20], so as to prevent or delay the selection of strong resistance. If CAPS testing finds that strong resistance alleles are present and/or in high frequency, then pest managers should consider stopping phosphine fumigation at that location in favor of alternative control methods, and thus not select for even stronger resistance [21]. The work reported here suggests that molecular markers for phosphine resistance could be used commonly in developing resistance management programs.

## Supporting Information

S1 Table Primers used for RT-PCR and molecular marker development. (DOCX)

## References

[1] Hagstrum DW, Phillips TW, Cuperus GW. Stored Product Protection. Kansas State University, KSRE Publ S–156; 2012.

[2] Tyler PS, Taylor RW, Rees DP. Insect resistance to phosphine fumigation in food warehouses in Bangladesh. Int Pest Cont. 1983;25:10–13, 21.

[3] Zettler JL, Cuperus GW. Pesticide resistance in *Tribolium castaneum* (Coleoptera: Tenebrionidae) and *Rhyzopertha dominica* (Coleoptera: Bostrichidae) in wheat. J Econ Entomol. 1990;83: 1677–1681.

[4] Rajendran S. Selection for resistance to phosphine or methyl bromide in *Tribolium castaneum* (Coleoptera, Tenebrionidae). Bull Entomol Res. 1992;82 **:** 119–124.

[5] Ren YL, O’Brien LG, Whittle GP. Studies on the effect of carbon dioxide in insect treatment with phosphine In: Highley E, Wright EJ, Banks H, Champ BR, editors. Stored Products Protection. Proceedings of the 6th International Conference on Stored Product Protection. CAB Press; 1994 pp. 173–177.

[6] Acda MA, Bengston M, Daglish GJ. Response to phosphine of susceptible and resistant strains of *Rhyzopertha dominica* . Life Sci. 2000;9:103–113.

[7] Lorini I, Collins PG, Daglish GJ, Nayak MK, Pavic H. Detection and characterization of strong resistance to phosphine in Brazilian *Rhyzopertha dominica* (F.) (Coleoptera: Bostrychidae). Pest Man Sci. 2007;63:358–364. 10.1002/ps.1344 17315137

[8] Opit GP, Phillips TW, Aikins MJ, Hasan MM. Phosphine resistance in *Tribolium castaneum* and *Rhyzopertha dominica* from Stored Wheat in Oklahoma. J Econ Entomol. 2012;105:1107–1114. 22928286 10.1603/ec12064

[9] Food and Agriculture Organization. Recommended methods for the detection and measurement of resistance of agricultural pests to pesticides. Tentative method for adults of some major pest species of stored cereals with methyl bromide and phosphine. FAO method no.16. FAO Plant Prot. Bull.1975;23:12–25.

[10] Collins PJ, Daglish GJ, Bengston M, Lambkin TM, Pavic H. Genetic resistance to phosphine in *Rhyzopertha dominica* (Coleoptera: Bostrichidae). J Econ Entomol. 2002;95:862–869. 12216832 10.1603/0022-0493-95.4.862

[11] Schlipalius DI, Cheng Q, Reilly TP, Collins PJ, Ebert PR. Genetic linkage analysis of the lesser grain borer, *Rhyzopertha dominica* identifies two loci that confer high-level resistance to the fumigant phosphine. Genetics. 2002;161:773–782. 12072472 10.1093/genetics/161.2.773 PMC1462159

[12] Schlipalius DI, Chen W, Collins PJ, Nguyen T, Reilly P, Ebert PR (2008) Gene interactions constrain the course of evolution of phosphine resistance in the lesser grain borer, *Rhyzopertha dominica* . Heredity. 2008;100:506–516. 10.1038/hdy.2008.4 18270533

[13] Jagadeesan R, Collins PJ, Daglish GJ, Ebert PE, Schlipalius DI. Phosphine resistance in the rust red flour beetle, *Tribolium castaneum* (Coleoptera: Tenebrionidae): inheritance, gene interactions and fitness costs. PLoS One 2012;7:1–12. 10.1371/journal.pone.0031582 PMC3283673 22363681

[14] Schlipalius DI, Valmas N, Tuck AG, Jagadeesan R, Ma L, Kaur R et al A core metabolic enzyme mediates resistance to phosphine gas. Science. 2012;338: 807–810. 10.1126/science.1224951 23139334

[15] Yeam I, Kang BC, Lindeman W, Frantz JD, Faber N, Jahn N. Allele-specific CAPS markers based on point mutations in resistance alleles at the pvr1 locus encoding eIF4E in Capsicum. Theor and Appl Gen. 2005;112:178–186. 16283234 10.1007/s00122-005-0120-2

[16] Kaur R, Daniels EV, Nayak MJ, Ebert PR, Schlipalius DI. Determining changes in the distribution and abundance of a *Rhyzopertha dominica* phosphine resistance allele in farm grain storage using a DNA marker. Pest Man Sci. 2013;69:685–688. 10.1002/ps.3514 23408750

[17] Katoh KK, Misawa K, Kuma K, Miyata T. MAFFT: a novel method for rapid multiple sequence alignment based on fast Fourier transform. Nucl Acids Res. 2002;30:3059–3066. 12136088 10.1093/nar/gkf436 PMC135756

[18] Sehgal B, Subramanyam B, Arthur F, Gill B. Variation in susceptibility of field strains of three stored-grain insect species to spinosad and chlorpyrifos-methyl plus deltamethrin on hard red winter wheat. J Econ Entomol. 2013;106:1911–1999. 24020310 10.1603/ec13083

[19] Daglish GJ, Collins PJ. Improving the relevance of assays for phosphine resistance In: Zuxun J., Quan L., Yongsheng L. Xianchang T and Langhua G. editors. Proceedings of the 7^th^ International Working Conference of Stored Product Protection. Sichuan Publishing House of Science and Technology; 1999 pp 584–593.

[20] Emery RN, Nayak MK, Holloway JC. Lessons learned from phosphine resistance monitoring in Australia. Stewart Postharvest Rev. 2011;7:1–8.

[21] Opit GP, Collins PJ Daglish GJ. Resistance management In: Hagstrum DW, Phillips TW, Cuperus GW, editors. Stored Product Protection. Kansas State University, KSRE Publ S–156. 2012 pp. 143–155.

