# Supplementary material for: Diagnostic Molecular Markers for Phosphine Resistance in U.S. Populations of Tribolium castaneum and Rhyzopertha dominica
Source: PLoS One. 2015 Mar 31;10(3):e0121343. doi: 10.1371/journal.pone.0121343 (PMC4380312; doi:10.1371/journal.pone.0121343)
Supplement: S1 Table — (DOCX) [file pone.0121343.s001.docx]

**S1 Table. Primers used for RT-PCR and molecular marker development^1^**

| Primer name | Primer sequence (5’-3’) | Product size (bp) | Usage |
| --- | --- | --- | --- |
| Tc-F  Tc-R | CGGAAAAAAATGGGCAGC  CACCGGGAGGTCATCATA | 1600 | Amplification of cDNA from Tc and sequencing |
| Tc-In-F  Tc-In-R | CTGACTTTAACAACTCCTCCCG  CGGTGTAATCGGCCTTGAAT |  | Sequencing |
| Rd-F  Rd-R | TCCCGGTCCAACATTTTAGTA  CGGCCTAACCTTAAAAATAC | 1800 | Amplification of cDNA from Rd and sequencing |
| Rd-In-F  Rd-In-R | AGGTTAGCGTGGAAGATGT  AGTTGACGGGAATGCAGC |  | Amplification of cDNA from Rd and sequencing |
| Tc-MM-F  Tc-MM-R | GCCCTGACTGTCTTCCACCA  AGCCTTGACAGCATTTTCCT | 368 | Molecular marker (MM) development in Tc |
| Rd-MM-F  Rd-MM-R | AGGTCCAAGCGTAGGGTTTT  AACTGGGAGAATTCGGCTTT | 375 | Molecular marker (MM) development in Rd |

^1^ All primers were the same as those used by Schlipalius et al. 2012 [14].
